# Early assessment of ventricular synchronization and function after left bundle-branch-area pacing with right bundle-branch block

**DOI:** 10.1186/s12872-022-02818-z

**Published:** 2022-08-21

**Authors:** Ruohan Zhao, Feng Xiong, Xiaoqi Deng, Shuzhen Wang, Chunxia Liu, Min Xu, Kunyue Tan, Xiuxiu Wang

**Affiliations:** Department of Cardiology, Cardiovascular Institute of Chengdu, Chengdu Third People’s Hospital/The Affiliated Hospital of Southwest Jiaotong University, No.82, Qinglong Street, Qingyang District, Chengdu, 610031 Sichuan Province China

**Keywords:** Left bundle-branch-area pacing, Right bundle-branch block, Two-dimensional speckle tracking imaging, Tissue mitral annular displacement, Right ventricular function

## Abstract

**Aim:**

To evaluate ventricular synchronization and function in patients with right bundle-branch block after left bundle-branch-area pacing (LBBAP) by echocardiography.

**Methods:**

Forty patients who successfully received LBBAP were selected and divided into the right bundle-branch block group (RBBB group) and the non-RBBB group by pre-operation ECG. Echocardiography and follow-up were performed 1 month after operation. Interventricular synchronization was evaluated by tissue Doppler (TDI), tissue mitral annular displacement (TMAD), and interventricular mechanical delay. The tricuspid annular plane systolic excursion (TAPSE), right ventricular fractional area change (RVFAC), tricuspid annulus sidewall systolic velocity (TV-s’), left ventricular global ventricular longitudinal strain (GLS), right ventricular free wall longitudinal strain (LS-RV), standard deviation of left ventricular 18 segments peak time difference (SDt-L) and standard deviation of right ventricular free wall 3 segments peak time difference (SDt-R) were applied to evaluate intraventricular synchronization and ventricular function.

**Results:**

The difference of displacement peak time of the tricuspid and mitral valves, namely ΔPT_TV-MV_ measured by TMAD, the difference of systolic time to peak of the tricuspid and mitral valves, namely ΔTs_TV-MV_ measured by TDI, were statistically different between the two groups (*P* < 0.05). Compared with the non-RBBB group, there were no statistically significant differences in the GLS, RVFAC, LS-RV, TAPSE, TV-s’, SDt-L, SDt-R (P > 0.05).

**Conclusion:**

Echocardiography technology including two-dimensional speckle tracking imaging (2D-STI), TDI, and TMAD can effectively analyze interventricular synchronization, intraventricular synchronization, and ventricular function. Although the movement of the right ventricular myocardium in the RBBB group was slightly later than that of the left ventricular myocardium after LBBAP, LBBAP could still be applied in RBBB patients with pacing indication.

**Supplementary Information:**

The online version contains supplementary material available at 10.1186/s12872-022-02818-z.

## Introduction

**Fig. 1:**
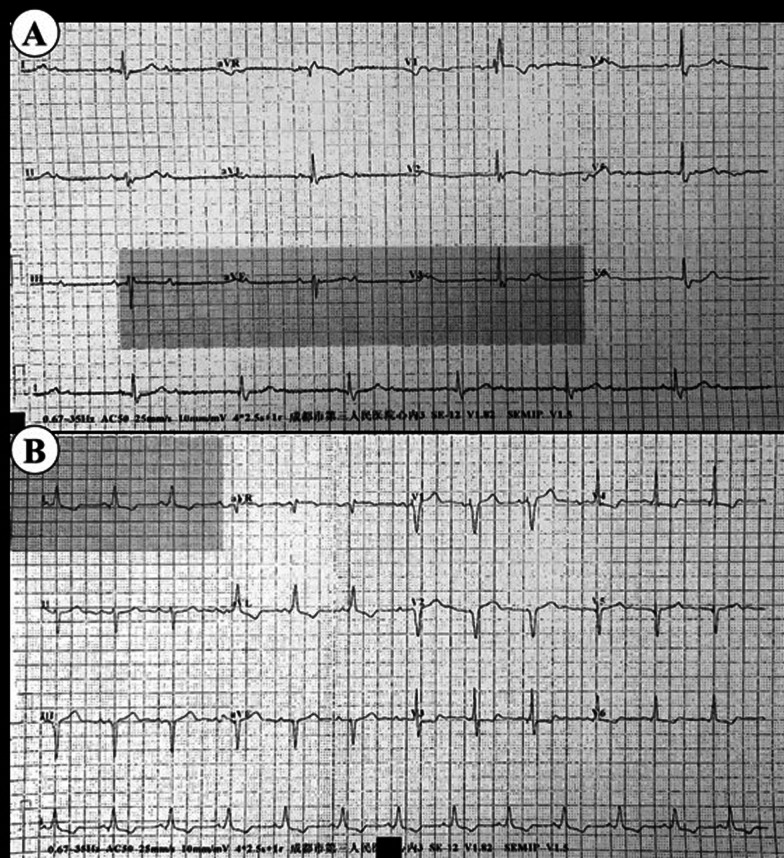
ECG of patients with RBBB before LBBAP (**A**) and after (**B**)

Left bundle-branch-area pacing (LBBAP) is a kind of physiological pacing with a low and stable pacing threshold [1]. After LBBAP, the patient’s ECG often shows a complete or an incomplete right bundle-branch block (RBBB) pattern [2, 3]. After optimizing the atrioventricular interval, right bundle branch conduction can merge with the pacing signals, so that RBBB morphology can be eliminated. However, for patients with intrinsic RBBB, the conduction of the right bundle branch is delayed or blocked. Although the duration of QRS after LBBAP is shorter than before, and the RBBB morphology of the electrocardiogram is improved(Fig. 1) [3, 4], there is still a difference in the optimal atrioventricular interval delay (AVD) between the RBBB and non-RBBB group[5]. On the premise of atrioventricular interval optimization, whether the RBBB will cause ventricular asynchrony and decline in ventricular function in RBBB patients after LBBAP has not been discussed yet. This study is conducted to explore the interventricular and intraventricular synchronization and ventricular function of RBBB patients after LBBAP, compared with non-RBBB patients.

## Materials and methods

### Subjects

The ethics committee of The Third People's Hospital of Chengdu approved to carry out the study within its facilities, and all methods were performed in accordance with the relevant guidelines and regulations. Informed consent was obtained from all subjects. This study was carried out in Chengdu Third People’s Hospital in patients who were indicated for pacing therapy according to 2013 ESC/EHRA Guideline. The criteria for exclusion were as follows: (1) Patients with left ventricular ejection fraction of less than 50%; (2) Patients with arrhythmia, such as atrial fibrillation; (3) Patients with congenital heart disease, valvular heart disease, and myocardial disease; (4) Unclear acoustic window. Patients completed an electrocardiogram to confirm whether they had RBBB and then were divided into the RBBB group or the non-RBBB group.

All 40 patients were implanted with dual-chamber pacemakers using Medtronic RELIA REDR 01 pulse generator. The atrial electrode was fixed in the right auricle, and the ventricular electrode (3830 electrode) was placed transseptally from the right ventricular septum to LV septal subendocardium in the LBB region with the assistance of C315 sheath. The potential of the left bundle branch could be recorded. ‘W’ pattern with the notch closer to nadir in lead V1 may indicate ideal location of left ventricular electrode. After testing the conventional parameters of the pacemaker (perception, threshold and impedance), we connected the pulse generator.

The pre-optimized AVD was determined empirically by the cardiologist. The post-optimized AVD was determined under the assistance of echocardiography. Firstly, the cardiologist set the pacemaker to DDD mode, and increase the AVD from 80 ms at the increment of 20 ms until 240 ms or intrinsic cardiac rhythm. Secondly, velocity time integral of aortic valve (VTI), LVEF, mitral anterior blood flow spectrum, mitral and tricuspid regurgitation are recorded at each AVD. Finally, the optimal atrioventricular phase (AVDopt) is determined mainly based on VTI, supplemented by the rest of the parameters. The partial parameters between AVDopt and different AVDs in patients with LBBAP were listed in the Additional file 1: Table S1.

### Image acquisition

Conventional echocardiography was performed by the Philips IE Elite color Doppler ultrasound diagnostic apparatus, equipped with an S5-1 probe, a frequency of 1–5 MHz, and a Qlab13 workstation. The conventional pre-operation echocardiography was performed in 3 days before the pacemaker implantation. One month after the implantation, the programmer set the pacing mode to the DDD unipolar pacing mode, and we optimized the atrioventricular interval according to LVEF, aortic velocity time integral, mitral regurgitation and tricuspid regurgitation. Under the optimal atrioventricular interval, the patient took the left side decubitus, synchronously connecting it to the electrocardiogram, and then performed image acquisition. To avoid beat-to-beat variance, the heartbeat fluctuation does not exceed 5 beats per min during the image acquisition. The specific operations were as follows: (1) conventional echocardiographic parameters measured according to the guideline of ASE[6]: left atrial diameter(LAd), left atrial volume index(LAVI), right atrial diameter (RAD), left ventricular end-diastolic diameter(LEVDd), left ventricular end-systolic Volume (LVESV), left ventricular end-diastolic Volume (LVEDV), left ventricular ejection fraction (LVEF) (pre-operation LVEF by M-Mode, post-operation LVEF by simpson’s method), right ventricular fractional area change(RVFAC); (2) We obtained the forward blood flow spectrum of the patient’s aortic valve and pulmonary valve; (3) The M-mode cursor was oriented to the junction of the tricuspid valve and the RV free wall in apical four-chamber view to get tricuspid annular plane systolic excursion (TAPSE); (4) We measured tricuspid annulus sidewall systolic velocity (TV-s’) by tissue Doppler (TDI); (5) We acquired apical four-chamber, three-chamber, and two-chamber images for three consecutive cardiac cycles, where the image completely contained the left and right ventricles, and the frame rate was > 50fps. (6) We acquired continuous acquisition of three cardiac cycles of the apical four-chamber TDI dynamic image.

### Image analysis

**Fig. 2:**
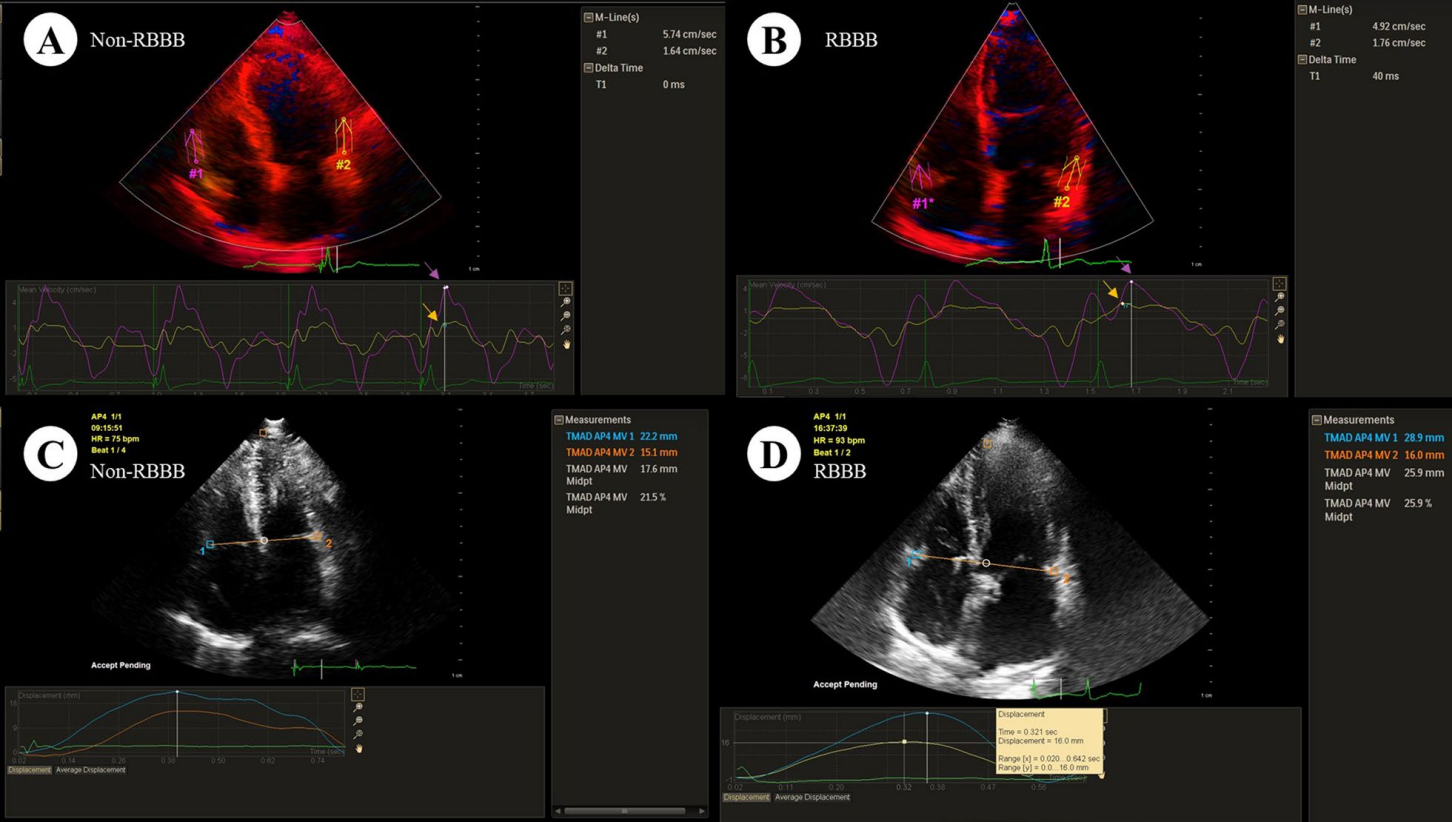
**A**, **B** TDI measurement of left and right ventricular lateral wall basal segment myocardial contraction velocity peak time, where A is non-RBBB, and B is RBBB. ΔTs_TV-MV_ in the RBBB group is greater than that of the non-RBBB group (arrows indicate the peak position); **C**, **D** TMAD measures the average PT of the mitral and tricuspid annulus sidewalls. **C** shows the non-RBBB group, and **D** shows RBBB. For the RBBB group, the peak time of the maximum displacement of the mitral and tricuspid valve annulus is quite different

**Fig. 3:**
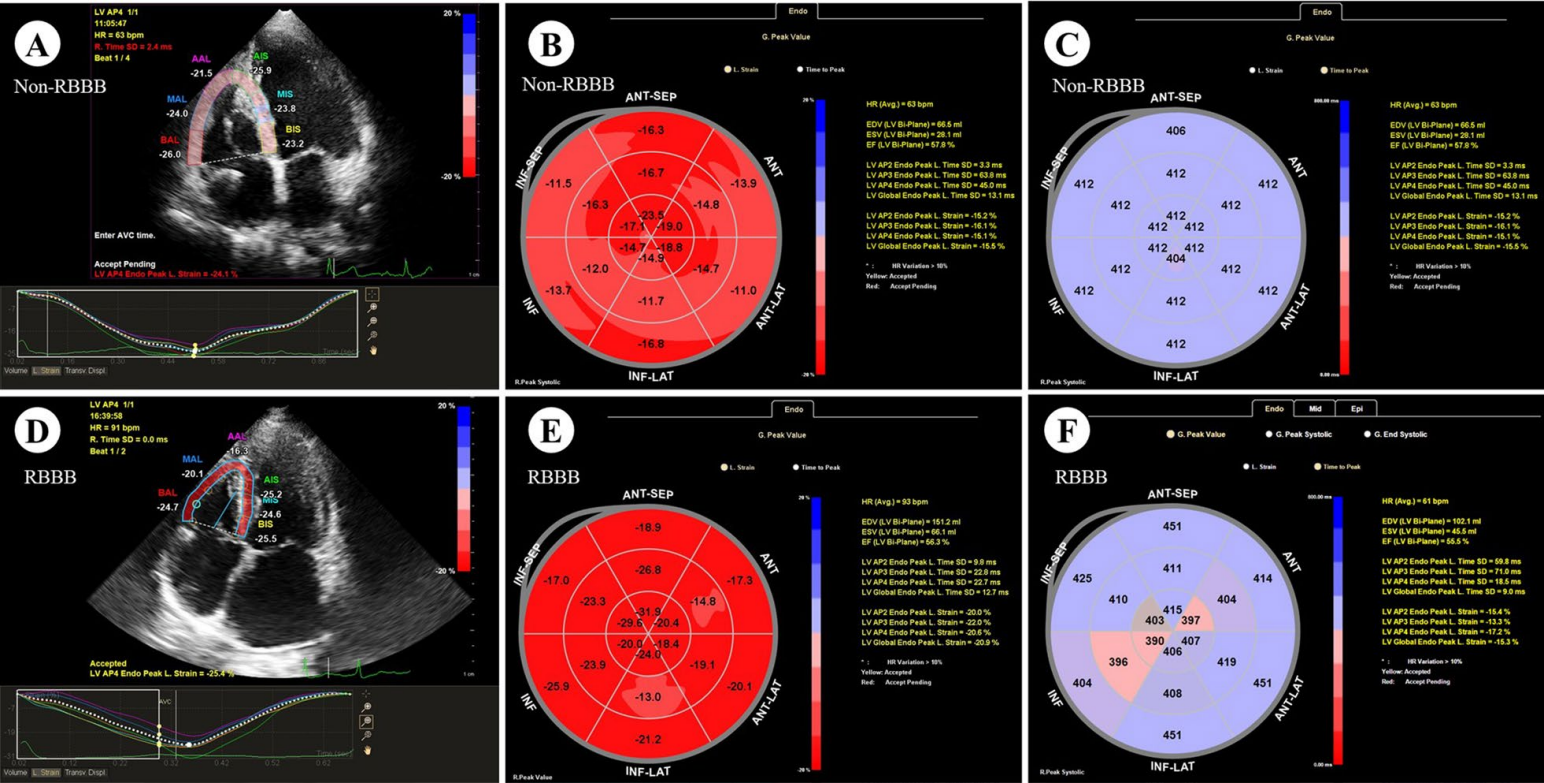
**A**, **D** 2D-STI measures the peak time of LS and LS of each segment of the right ventricle, **A** is non-RBBB, **D** is RBBB; **B**, **E** LS of each segment of the left ventricle, B is non-RBBB, **E** is RBBB; **C** **F** The peak time of LS of each segment of the left ventricle, **B** is non-RBBB, **E** is RBBB. There is no difference between the two groups

We used echocardiographic technique, such as blood flow spectrum, 2DQ and TDI to evaluate interventricular synchronization. The parameters were measured as follows: (1) We measured the time from the beginning of QRS to the beginning of the blood flow spectrum on the pulmonary valve namely pulmonary pre-ejection interval (PPEI), and the time from QRS to the beginning of the blood flow spectrum on the aortic valve, namely artery pre-ejection interval (APEI). The difference of PPEI and APEI is IVMD; (2) We selected the apical four-chamber TDI dynamic image and entered the SQ plug-in, outlined the tricuspid valve (TV) sidewall and mitral valve (MV) sidewall, and obtained the myocardial motion curve of the right ventricular basal segment and the left ventricular basal segment. The difference of systolic time to peak of TV and MV were recorded as ΔTs_TV-MV_ (Fig. 2A, B); (3) We selected apical four-chamber two-dimensional dynamic image and entered TMAD mode. Then, we placed the fixed points on the TV sidewall, MV sidewall and the left ventricular apex respectively. The software would automatically generate two simultaneous displacement curves of sampling points. The difference of the displacement peak time (PT) of the MV and TV are recorded as ΔPT_TV-MV_ (Fig. 2C, D).

The evaluation of intraventricular systolic synchronization and systolic function was as follows: Two-dimensional speckle tracking imaging (2D-STI) of the Qlab 13 workstation was used to analyze the left ventricular global longitudinal strain (GLS) and longitudinal strain of right ventricular free wall (LS-RV) (Fig. 3A, D) to reflect the ventricular function. The right ventricle was partitioned into 6 standard segments at 3 levels (i.e., the basal, middle, and apical levels), correspondingly generating 6 time-strain curves. LS-RV was evaluated in the basal, midventricular, and apical segments of the RV free wall and calculated as the average of the 3 segments. The standard deviation of systolic peak time was calculated to reflect the asynchrony index of the left (SDt-L) and right ventricles (SDt-R) to reflect the intraventricular synchronization.

### Reproducibility

Of the 40 LBBBAP patients, 16 patients were selected randomly to evaluate the reproducibility of IVMD, TMAD, TDI and 2D-STI. For intra-observer variability, analyses were repeated by the same primary investigator. For inter-observer variability, analyses were performed by 2 masked investigators. During the repeated analyses, investigators were masked to the results of the first measurements.

### Statistical methods

SPSS23.00 software was used for statistical analysis. Continuous variables with normal distribution are presented as the mean ± standard deviation. Continuous variables with non-normal distribution are presented as median (interquartile range). Categorical variables are expressed as percentages. The differences between two groups were assessed with the chi-square analysis for categorical variables and the t-test or non-parametric tests for continuous data at baseline. P < 0.05 was considered statistically significant.

## Results

### General situation analysis

A total of 40 patients who successfully underwent left bundle-branch pacing were included in this study, including 22 males and 18 females. The average age of the enrolled patients was (70.88 ± 12.95) years; there were 26 patients with hypertension, 11 patients with diabetes, and 6 with coronary heart disease. Moreover, for the indication of pacemaker implantation, 10 patients are owing to high-grade atrioventricular block, 19 patients owing to third-degree atrioventricular block, 11 patients owing to a second-degree type II atrioventricular block. Among the non-RBBB group, there are 7 patients with LBBB, 14 patients with normal QRS. After LBBAP treatment, the pacing parameters were satisfied: The ventricular capture is at low output (< 1.5 V/0.5 ms), and the impedance is > 500 Ω by unipolar pacing. According to the preoperative electrocardiogram, the patients were divided into two groups, including 19 patients in the RBBB group and 21 patients in the non-RBBB group. The optimal AVD in RBBB group was shorter than those without right bundle branch block (*P* < 0.05). There was no statistical difference between the two groups in terms of age, gender, comorbidities, etiology, pacemaker parameters, or preoperative cardiac color Doppler ultrasound parameters (*P* > 0.05). See Table 1 for details.

**Table 1:** General condition of the patient

Parameters	Total (n = 40)	RBBB group (n = 19)	Non-RBBB group (n = 21)	*P*
Age/years	70.88 ± 12.95	74.71 ± 9.68	69.39 ± 14.00	0.37
Male/n (%)	22 (55%)	8 (42.1%)	14 (66.7%)	0.08
Complication/n (%)				
Hypertension	26 (65%)	12 (63.2%)	14 (67.7%)	0.17
Diabetes	11 (28%)	5 (26.3%)	6 (28.6%)	0.28
Coronary heart disease	6 (15%)	2(10.5%)	4 (19.0%)	0.69
Pathogen/n (%)				
High-grade atrioventricular block	10 (25%)	6 (28.6%)	4(19.0%)	0.71
Third degree atrioventricular block	19 (48%)	8 (42.0%)	11 (52.3%)	
Second degree type II atrioventricular block	11 (28%)	6 (31.6%)	5 (23.8%)	
Ventricular perception/mV	10.95 ± 5.43	9.53 ± 3.25	11.58 ± 6.13	0.42
Ventricular capture/V	0.60(0.40–0.90)	0.60(0.40–0.80)	0.60(0.43–1.05)	0.62
Impedance/Ω	752.96 ± 214.89	818.14 ± 234.76	724.44 ± 206.96	0.35
Pre-operation				
LAd/mm	38.16 ± 5.23	39.43 ± 5.74	37.67 ± 5.09	0.46
Right atrium transverse diameter/mm	38.40 ± 3.89	39.43 ± 3.82	38.00 ± 3.95	0.42
Right atrium vertical diameter/mm	46.96 ± 4.45	48.29 ± 4.86	46.44 ± 5.41	0.37
LVEDd/mm	46.16 ± 4.82	45.43 ± 5.41	46.44 ± 4.71	0.65
LVEF/%	60.08 ± 4.13	60.57 ± 2.51	59.89 ± 4.66	0.72
QRSd/ms^a^	112.94 ± 26.05	136.00 ± 12.83	104.08 ± 24.49	0.02*
Post-operation				
LAVI/ml/m2	22.74 ± 9.26	21.86 ± 9.23	23.13 ± 9.55	0.77
Right atrium transverse diameter/mm	38.4 ± 4.00	38.71 ± 4.19	38.27 ± 4.04	0.81
Right atrium vertical diameter/mm	48.08 ± 4.7	49.00 ± 4.93	47.72 ± 4.70	0.55
LVEDVI/ml	37.99 ± 10.11	38.25 ± 13.65	38.25 ± 8.68	0.94
LVESVI/ml	16.18 ± 5.40	16.89 ± 5.47	15.86 ± 5.51	0.68
LVEF/%	58.84 ± 5.07	60.00 ± 3.74	58.39 ± 5.53	0.49
QRSd/ms^a^	124.59 ± 11.31	135.20 ± 10.78	120.17 ± 8.45	0.01*
Pre-optimized AVD/ms^b^	145.71 ± 14.53	132.00 ± 17.89	151.43 ± 15.11	0.03*
Post-optimized AVD/ms^b^	151.43 ± 15.11	131.43 ± 15.74	153.61 ± 16.79	0.01*

^*^RBBB and non-RBBB group is statistically different in these aspects

^a^QRS is not statistically different before and post the operation

^b^AVD is not statistically different before and post the optimization

### TMAD, IVMD, and TDI assess left and right ventricular synchrony

There was no significant difference in IVMD between the two groups (*P* > 0.05). ΔTs_TV-MV_ between two groups has statistical difference (*P* < 0.05). The ΔTs_TV-MV_ of RBBB group and non-RBBB group were (47.29 ± 58.45) ms, (− 12.00 ± 49.91) ms respectively. ΔPT_TV-MV_ measured was statistically different between two groups (*P* < 0.05). Moreover, the RBBB group was (28.14 ± 39.04) ms, and the non-RBBB Group was (− 28 ± 48.26) ms. Both the difference of ΔTs_TV-MV_ and ΔPT_TV-MV_ showed that in RBBB group the tricuspid side segment activated slower than the mitral side. See Table 2 for details.

**Table 2:** TMAD, IVMD and TDI to assess the synchrony of the left and right ventricles

Parameters	RBBB group	Non-RBBB group	*P*
APEI/ms	185.2 ± 80.63	117.70 ± 11.88	0.02*
PPEI/ms	177.60 ± 81.52	117.40 ± 20.09	0.04*
IVMD/ms	7.60 ± 6.77	0.20 ± 20.44	0.45
PT_TV_/ms	355.29 ± 71.12	385.25 ± 78.22	0.40
PT_MV_/ms	327.14 ± 77.15	413.25 ± 73.78	0.02*
ΔPT_TV-MV_/ms	28.14 ± 39.04	− 28.00 ± 48.26	0.01*
ΔTs_TV-MV_/ms	47.29 ± 58.45	− 12.00 ± 49.91	0.02*

^*^RBBB and non-RBBB group is statistically different in these aspects

### Ventricular systolic synchronization and systolic function

Compared with the non-RBBB group, the LVEF, GLS, RVFAC, LS-RV, TAPSE, TV-s’, SDt-L, SDt-R in the RBBB group were not statistically different (*P* > 0.05) (Fig. 3). See Table 3 for details.

**Table 3:** Assessment of ventricular synchronization and ventricular function

Parameter	RBBB group	Non-RBBB group	P
LV			
LVEF/%	60.00 ± 3.74	58.39 ± 5.53	0.49
GLS/%	− 19.00 (− 20.00 to − 18.00)	− 18.00 (− 19.00 to − 15.25)	0.64
SDt-L/ms	50.00 (36.00–65.00)	24.00 (20.00–36.75)	0.13
RV			
TAPSE/mm	16.33 ± 1.15	19.27 ± 2.24	0.06
TV-s'/cm/s	13.86 ± 3.39	13.19 ± 2.86	0.63
RVFAC/%	49.86 ± 7.37	52.37 ± 8.05	0.49
LS-RV/%	− 20.57 ± 10.21	− 19.65 ± 12.50	0.87
SDt-R/ms	65.43 ± 40.34	66.56 ± 37.22	0.95

### Reproducibility

The measurement of IVMD, TMAD, TDI and 2D-STI showed good reproducibility. The complete data of inter-observer variability and intra-observer variability were shown in Table 4.

**Table 4:** Inter-Observer and Intra-Observer reproducibility of the study

	ICC (95%CI)
Inter-observer (n = 16)	
IVMD	0.98 (0.95, 0.99)
ΔPT_TV-MV_	0.99 (0.95, 0.99)
ΔTs_TV-MV_	0.92 (0.92, 0.99)
LVEF	0.80 (0.50, 0.93)
GLS	0.80 (0.51, 0.93)
SDt-L	0.99 (0.98, 0.99)
RVFAC	0.90 (0.20, 0.98)
LS-RV	0.90 (0.72, 0.96)
SDt-R	0.96 (0.88, 0.99)
Intra-observer (n = 16)	
IVMD	0.91 (0.72, 0.97)
ΔPT_TV-MV_	0.97 (0.93, 0.99)
ΔTs_TV-MV_	0.92 (0.78, 0.97)
LVEF	0.83 (0.56, 0.94)
GLS	0.90 (0.50, 0.97)
SDt-L	0.92 (0.64, 0.98)
RVFAC	0.85 (0.63, 0.95)
LS-RV	0.76 (0.44, 0.91)
SDt-R	0.97 (0.91, 0.99)

## Discussion

The right bundle branch originates from the His bundle and divides into three branches at the base of the tricuspid anterior papillary muscle. It runs along the low part of the ventricular septum, the anterior wall of the right ventricle, the free wall of the right ventricle, the posterior papillary muscle, and the lower right posterior part of the ventricular septum. The electrical excitement is quickly transmitted to each segment of the right ventricular wall through the three branches, ensuring the interventricular and intraventricular synchronous contraction. However, because the right bundle branch is slender, superficial, mostly supplied by a single branch of the left anterior descending branch, the right bundle-branch conduction system is prone to conduction disorders. Therefore, RBBB is common in clinical practice, with an incidence of 8%, which increases with age [7, 8]. Herein, the incidence of RBBB in patients undergoing pacemaker implantation is not supposed to be low. The optimal pacing method for bradyarrhythmia with RBBB remains to be explored. His-Bundle pacing, cardiac resynchronization therapy, and right bundle-branch pacing can correct RBBB morphology, but it has the disadvantages of complicated operation process, unstable pacing threshold, and low success rate [9–11]. At present, LBBAP is a physiological pacing method with high success rate, stable pacing threshold for bradyarrhythmia. A case report showed that LBBAP could eliminate the RBBB morphology in patient with RBBB [12]. However, there is currently a lack of echocardiographic techniques to study the ventricular function and synchronization of RBBB patients after LBBAP.

**Figure4:**
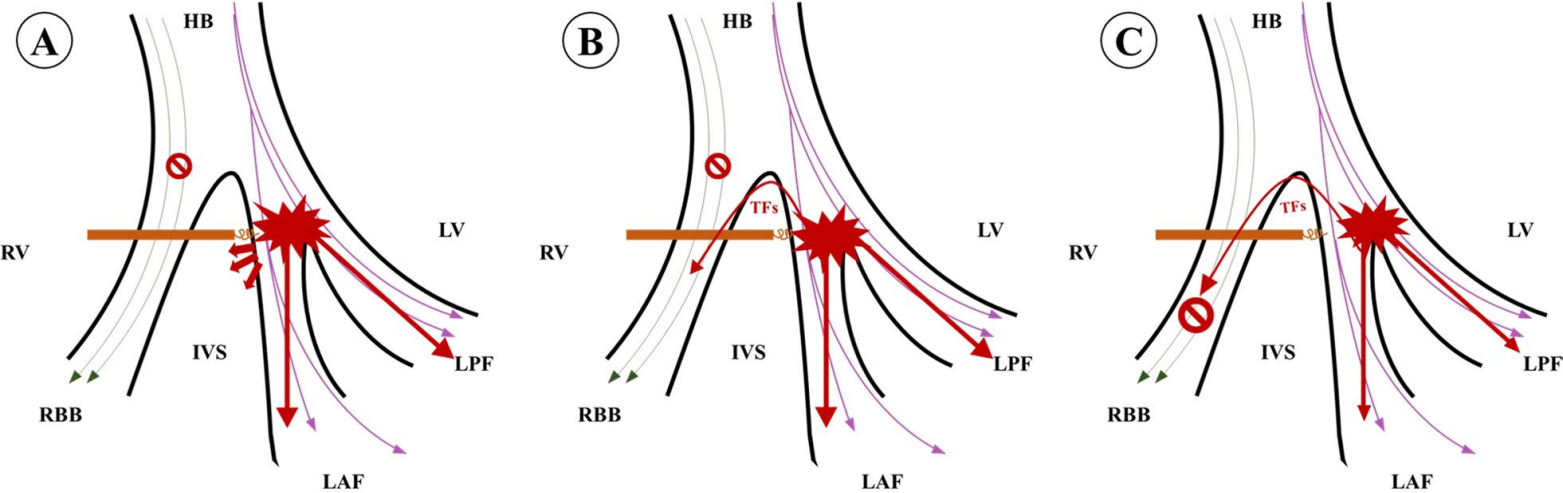
**A** When accompanied by RBBB, LBBAP stimulates the right ventricular myocardial cells in the left bundle-branch area, and the right ventricle is activated through intercellular conduction; **B** When RBBB is located in front of TFs, the left ventricular pacing signal is transmitted to the right bundle through TFs. **C** When the RBBB is located at the distal end of the TFs, the pacing signal of the left ventricle is not always transmitted to the right ventricle through the TFs to achieve physiological pacing of the right ventricle. (HB: His bundle; RBB: right bundle branch; LAF: left anterior branch; LPF: left posterior branch; RV: right ventricle; IVS: interventricular septum; LV: left ventricle)

In this study, we performed LBBAP on patients with and without RBBB who had no statistical difference in general conditions. In the beginning, we found a difference in the optimal AVD between the RBBB and non-RBBB group, which is in accord with our former study[5]. After that, we used the IVMD, TMAD, and TDI to evaluate the synchronization of left and right ventricular myocardial movements. Through these three methods, we found the followings: (1) The movement of the right ventricular myocardium in the RBBB group after LBBAP treatment was slightly later than that of the left ventricular myocardium; (2) TMAD and TDI techniques are more likely to detect the interventricular desynchrony than IVMD. In this study, we used unipolar LBBAP pacing. There are two possibilities for pacing signals to be transmitted to the right ventricle: (1) The right ventricular myocardium in the region of the left bundle branch is stimulated, and the right ventricle is stimulated through intercellular conduction. This conduction method may cause asynchrony between the right and left chambers (Fig. 4A); (2) In previous studies, the existence of interconnection fibers (TFs) between the left and right bundle branches was also proposed. The pacing signal of the left ventricle may be transmitted to the right bundle branch through TFs, which in turn stimulates the right ventricle physiologically (Fig. 4B) [12]. However, in the presence of RBBB, the pacing signal of the left ventricle may not be able to transmit to the right ventricle through TFs to achieve physiological pacing of the right ventricle (Fig. 4C), so the synchronization of the two ventricles will be inconsistent. This argument was verified by our study. By the application of TMAD and TDI, we detected a slight difference between RBBB group and non-RBBB group. The maximum systolic displacement time and peak time of systolic velocity of TV side segment is slower than that of MV in RBBB group in the same cardiac cycle. The reasons why IVMD did not find the discrepancy are probably as follows: (1) PPEI and APEI come from different cardiac cycles, which is an important cause of error. (2) IVMD evaluates biventricular synchronization by hemodynamic method. While TMAD and TDI directly evaluate the mechanical myocardial synchronization of the ventricles. We supposed that the desynchrony of biventricular myocardial movement happen before the biventricular hemodynamics. Through this study, we found that patients with RBBB still had asynchrony of left and right ventricular contractions after LBBAP.

According to the study by Junyu et al. [13], SDt-R of complete RBBB is different from that of the control group, with 49.89 ± 4.79 ms versus 8.90 ± 1.67 respectively. Compared with this study, our data did not find a distinct difference in right ventricular asynchrony in two groups. Nor did we find difference in two groups in the aspect of right ventricular function, namely TAPSE, TV-s’, RVFAC and LS-RV, as well as in the aspect of left ventricular function and intraventricular asynchrony, namely LVEF, GLS and SDt-L. It suggests that at least RBBB did not exacerbate the asynchrony and dysfunction of right ventricle after LBBAP. A lot of researches have testified that LBBAP could ensure the physiological pacing of the left ventricle [14–20]. The morphology and function of the left and right ventricles affect each other. Insufficiency or asynchronization of the left heart can cause changes in the pulmonary artery pressure due to the increase in left atrial pressure, leading to an increase in the afterload of the right heart; Insufficiency or asynchrony of the right heart can cause the right heart to enlarge. In the limited volume of the pericardium, the enlarged right heart causes the diastolic restriction of the two ventricles. Studies have shown that right ventricular dysfunction or asynchrony of exercise is an early warning indicator of poor prognosis for heart failure and non-response of CRT [21, 22]. Herein, in the selection of pacing strategy, the function and synchronization of the two ventricular should all be taken into account. In the LBBAP patient, intrinsic RBBB does not impair right ventricular synchronization or function. Thus, LBBAP could be applied in RBBB patient to realize left ventricular physiological pacing. From this early assessment, we deduce that LBBAP could be safely applied in the patients with RBBB.

This study also had certain limitations. Firstly, the small sample size may have caused some errors in statistics. Secondly, because the right ventricle has an irregular crescent shape, we only studied the strain of the right ventricle in the apical four chambers; Thirdly, the detailed preoperative ultrasonic data of patients was not collected, so the changes of preoperative and postoperative ultrasound parameters of patients could not be analyzed; Finally, the follow-up time was short, so this study could only reflect the short-term impact of LBBAP on right ventricular function and synchronization. With a prolonged implantation time, whether the mechanical and electrical remodeling of the heart will have a long-term improvement effect on RBBB remains unknown. Whether it can reduce atrial fibrillation and cardiovascular adverse events caused by RBBB [8, 23] also remains to be further studied.

## Conclusion

In summary, after LBBAP, RBBB patients had a certain degree of asynchrony of left and right ventricular movements, but postoperative RBBB patients’ right ventricular synchronization and ventricular longitudinal strain were not statistically significant compared with the non-RBBB group. It indicates that LBBAP is an optional pacing strategy for RBBB patients with pacing indications.

## Supplementary Information

**Additional file 1: Table S1.** Comparison of parameters between AVDopt and different AVDs in patients with LBBAP

## Data Availability

The datasets generated and/or analyzed during the current study are not publicly available due to the ongoing project but are available from the corresponding author on reasonable request.

## Abbreviations

LBBAP: Left bundle-branch-area pacing
RBBB: Right bundle-branch block
AVD: Atrioventricular interval delay
TMAD: Tissue mitral annular displacement
IVMD: Interventricular mechanical delay
2D-STI: Two-dimensional speckle tracking imaging
TDI: Tissue Doppler
TAPSE: Tricuspid annular plane systolic excursion
TV-s’: Tricuspid annulus sidewall systolic velocity
RVFAC: Right ventricular fractional area change
LS-RV: Right ventricular free wall longitudinal strain
SDt-R: Standard deviation of right ventricular free wall 3 segments peak time difference
GLS: Left ventricular global longitudinal strain
SDt-L: Standard deviation of left ventricular 18 segments peak time difference
PPEI: Pulmonary pre-ejection interval
APEI: Artery pre-ejection interval
PT: Displacement peak time
ΔTs_TV__-MV_: The difference of systolic time to peak of TV and MV
ΔPT_TV__-MV_: Difference of PT_MV_ and PT_TV_
LAVI: Left atrial volume index
RAD: Right atrial diameter
LVESV: Left ventricular end-systolic Volume
LVEDV: Left ventricular end-diastolic Volume
LVEF: Left ventricular ejection fraction
TV: Tricuspid valve
MV: Mitral valve
TFs: Interconnection fibers
HB: His bundle
RBB: Right bundle branch
LAF: Left anterior branch
LPF: Left posterior branch
RV: Right ventricle
IVS: Interventricular septum
LV: Left ventricle
