# Supplementary material for: Early assessment of ventricular synchronization and function after left bundle-branch-area pacing with right bundle-branch block
Source: BMC Cardiovasc Disord. 2022 Aug 21;22:380. doi: 10.1186/s12872-022-02818-z (PMC9394046; doi:10.1186/s12872-022-02818-z)
Supplement: Supplementary file 1 — Additional file 1: Table S1. Comparison of parameters between AVDopt and different AVDs in patients with LBBAP [file 12872_2022_2818_MOESM1_ESM.pdf]

Table S1 Comparison of parameters between AVDopt and different AVDs in patients with LBBAP

	AVDopt	100ms	120ms	140ms	160ms	180ms	200ms	220ms
LVEF （%）	58.84±7.18	55.59±5.11	58.84±7.18	57.41±5.98	58.4±7.12	58.99±6.43	57.49±5.26	55.26±7.39
VTI （cm）	29.14±8.92	27.79±8.53	28.96±9.80	28.77±9.48	28.7±9.34	28.48±8.92	25.55±4.22	28.27±10.79
IVMD （ms）	0.56±16.35	43.47±194.72	3.63±15.73	1.56±18.39	3.31±17.56	2.47±17.11	0.36±18.02	2.18±18.54
LVFT/RR	0.46±0.08	0.48±0.05	0.47±0.69	0.46±0.08	0.45±0.08	0.43±0.09	0.38±0.09	0.38±0.10
P1	/	0.13	0.55	0.53	0.86	0.44	0.60	0.20
P2	/	0.65	0.95	0.91	0.89	0.83	0.22	0.81
P3	/	0.38	0.59	0.73	0.52	0.62	0.98	0.69
P4	/	0.29	0.62	0.88	0.88	0.38	0.03*	0.03*

PS: P1 represents the LVEF comparison of AVDopt and AVD in the current column. P2 represents the VTI comparison of AVDopt and AVD in the current column; P3 represents the IVMD comparison between AVDopt and AVD in the current column. P4 represents LVFT/RR comparison of AVDopt and AVD in the current column.

* Two groups are statistically different.
